# Anatomical Variations of the Human Cochlea Using an Image Analysis Tool

**DOI:** 10.3390/jcm12020509

**Published:** 2023-01-08

**Authors:** Raabid Hussain, Attila Frater, Roger Calixto, Chadlia Karoui, Jan Margeta, Zihao Wang, Michel Hoen, Herve Delingette, François Patou, Charles Raffaelli, Clair Vandersteen, Nicolas Guevara

**Affiliations:** 1 Research & Technology, Oticon Medical, 06220 Vallauris, France; 2 Clinical Evidence Department, Oticon Medical, 06220 Vallauris, France; 3 Research and Development, KardioMe, 01851 Nova Dubnica, Slovakia; 4 Epione Team, Inria, Université Côte d’Azur, 06902 Sophia Antipolis, France; 5 Institut Universitaire de la Face et du Cou, Nice, Centre Hospitalier Universitaire de Nice, Université Côte d’Azur, 06100 Nice, France

**Keywords:** cochlear morphology, cochlear implantation, statistical analysis

## Abstract

Understanding cochlear anatomy is crucial for developing less traumatic electrode arrays and insertion guidance for cochlear implantation. The human cochlea shows considerable variability in size and morphology. This study analyses 1000+ clinical temporal bone CT images using a web-based image analysis tool. Cochlear size and shape parameters were obtained to determine population statistics and perform regression and correlation analysis. The analysis revealed that cochlear morphology follows Gaussian distribution, while cochlear dimensions A and B are not well-correlated to each other. Additionally, dimension B is more correlated to duct lengths, the wrapping factor and volume than dimension A. The scala tympani size varies considerably among the population, with the size generally decreasing along insertion depth with dimensional jumps through the trajectory. The mean scala tympani radius was 0.32 mm near the 720° insertion angle. Inter-individual variability was four times that of intra-individual variation. On average, the dimensions of both ears are similar. However, statistically significant differences in clinical dimensions were observed between ears of the same patient, suggesting that size and shape are not the same. Harnessing deep learning-based, automated image analysis tools, our results yielded important insights into cochlear morphology and implant development, helping to reduce insertion trauma and preserving residual hearing.

## 1. Introduction

Cochlear implants (CI) are the most successful neural prosthetic devices to date that provide hearing to profoundly hearing-impaired people around the world. CIs work by bypassing hair cell functionality and applying electrical stimulation to the auditory nerve fibers directly via a multichannel electrode array ideally implanted in the scala tympani (ST). Among other factors, CIs were shown to provide better hearing outcomes, e.g., word recognition scores for patients with greater neural survival [1,2]. In recent years, patients with low-frequency residual hearing also became eligible for CIs [3,4], and the CIs have shown a superior performance compared to those in profoundly deaf users [5,6,7,8,9]. Preservation of neural structures and residual hearing is therefore of high importance as it can provide additional auditory cues and improve speech understanding. There are several factors that can affect the preservation of residual hearing during cochlear implant surgery. These include the surgical approach, the type of cochlear implant being used and the skill of the surgeon. Soft surgery, with its smaller incisions and less invasive approach, may be more likely to preserve residual hearing compared to traditional surgery [10]. However, each patient’s situation is unique, and the best approach for preserving residual hearing will depend on the individual’s specific needs and circumstances. The delicate process of CI electrode insertion is nevertheless prone to introducing damage to cochlear structures [11,12,13]. Cochlear damage was shown to relate to long-term neural degeneration [14,15] and was also associated with the loss of residual hearing [16,17,18,19].

Cochlear damage due to electrode insertion may be mitigated by less traumatic surgical procedures [20,21] and by the improvement of CI electrode array designs [22,23,24]. Manufacturers may offer electrode arrays that best match the needs of individuals by providing electrodes with different dimensions that are the most suitable for the candidates. However, cochlear size and morphology are known to have large inter-individual variability [25,26,27]. To guide electrode development, detailed information is required about the variability of parameters that describe the cochlear size and shape [27]. These parameters can be obtained from computed tomography (CT) images, which are routinely available from CI candidates [28].

Recent studies relating cochlear morphology to CI electrode insertion focused on the establishment of normative datasets and reliable cochlear size measures [28], quantification of internal cochlear dimensions with high precision [27], evaluation of electrode mechanical properties in relation to induced cochlear trauma [29] or the establishment of a mathematical model that describes the shape of the cochlea [30]. In this study, variability and correlation of cochlear parameters, extracted via 3D reconstruction by the Oticon Medical Nautilus software [31], are investigated in a large set of 1099 cochleae. Additionally, intra-patient, inter-patient and inter-sex similarities are also analyzed.

## 2. Materials and Methods

### 2.1. Dataset

A total of 590 patients undergoing various treatments at the Institut Universitaire de la Face et du Cou, Nice, France, from 2008 to 2013 were included in this retrospective study. Preoperative temporal bone CT scans were obtained for each patient, constituting a dataset of 1099 CT images comprising 560 right and 539 left scans. The acquired images were of varying quality, with voxel resolutions ranging from 0.187 × 0.187 × 0.250 to 0.316 × 0.316 × 0.312 mm^3^. In accordance with the data agreement, EU General Data Protection Regulation (GDPR) and local regulations on data privacy and processing, the dataset was fully pseudonymized before further processing. Therefore, the correspondence between the identifying metadata and the pseudonyms used to identify individual datasets was not available, with the exception of patients’ sex. Analysis related to patients’ demographics such as origin, age, etc., was beyond the scope of this study.

### 2.2. Image Analysis

A cooperative multi-agent reinforcement learning framework (C-MARL) as described in [32] was used to automatically detect the cochlear apex, center and round window landmarks for each image. Although only the cochlear center landmark was required for further processing, using a three landmark C-MARL approach ensured better detection of the landmark [33]. CT image-detected cochlear center landmark coordinates, cochlear side and operative status for each CT image were compiled and uploaded to Nautilus (v20220801; Oticon Medical, Vallauris, France)—a web-based cochlear image analysis tool [31].

**Figure 1:**
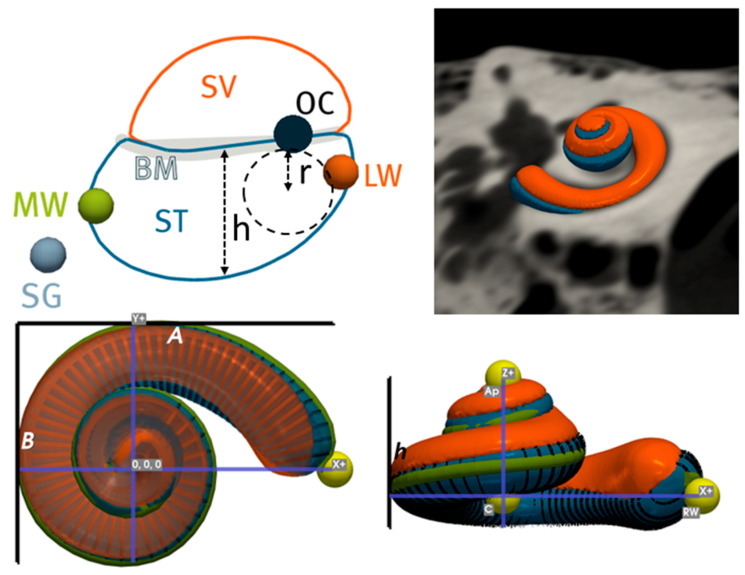
Description of clinical metrics computed by Nautilus. A: maximum length between round window and lateral wall; B: maximum perpendicular length to A. h: height of cochlea; h: maximum vertical ST height; r: radius of maximum circle that can fit in ST; ST: scala tympani; SV: scala vestibuli; BM: basilar membrane; OC: organ of corti; LW: lateral wall; MW: modiolar wall.

Nautilus processed the images automatically, generating the cochlear view, intracochlear segmentations and various clinically relevant cochlear parameters. Figure 1 depicts different parameters that Nautilus extracts from each image. Once all the images had been processed, an export bundle was prepared with the following characteristics for analysis: cochlear and ST models, cochlear size, shape, duct lengths and cross-sectional measurements. Nautilus’ output confidence scores were also exported and used to filter out any processing failures.

### 2.3. Statistical Analysis

A histogram of the cochlear parameters extracted by Nautilus such as volume, A, B, height, lateral wall (LW) length, the wrapping factor and roller coaster height was generated using 50 bins. Based on the mean and the standard deviation of the parameters, Gaussian curves were plotted on top of the histograms. Correlation analysis was performed via visual inspection of scatter plots and the calculation of the Pearson correlation coefficient between the aforementioned parameters, as well as between parameters A, B and the LW length at various cochlear angles. A regression curve was fitted to the correlation data by the ordinary least squares method. For the correlation and regression analysis, the relevant functions from the SciPy and Scikit-learn python packages were used [34,35].

Analysis of ST height, area and radius was performed up to a cochlear angle of 705°. The mean, standard deviation, 10th and 90th percentile of the ST angular data were calculated based on the data points falling within ±15° of every 30° ST angle, e.g., the metadata at 90° were based on individual data points between 75° and 105°.

Additionally, an intra-patient analysis was conducted to determine the similarity between contralateral ears. Four hundred fifty-eight patients for whom CT imaging was conducted for both ears were selected for the analysis. The ears were assessed with respect to both imaging and clinical metrics. For imaging analysis, the 3D left–right segmentation meshes were registered together based on landmarks [36,37]. Intra-patient Dice coefficients, Hausdorff distances and average symmetric surface distances were computed [38]. An inter-patient analysis was also conducted in which 18 patients were uniformly and randomly selected from the dataset and compared with all other patients (*n* = 440) in the dataset. Global metrics defining cochlear size and shape such as A, B, volume and duct lengths were also evaluated. Statistical *t*-tests with Holm–Sidak correction were performed to analyze the results. A *p*-value of <0.05 was considered significant. A correlation analysis was also performed to determine the relationship between different parameters.

Inter-sex comparison was also carried out based on the cochlear parameters generated by Nautilus. Both size and shape parameters were analyzed to gain insights into whether a distinction could be observed between both sexes. An independent two-sample *t*-test was conducted to determine whether the difference was significant. A *p*-value of <0.05 was considered significant.

## 3. Results

### 3.1. Population Statistics and Correlation Analysis

**Figure 2:**
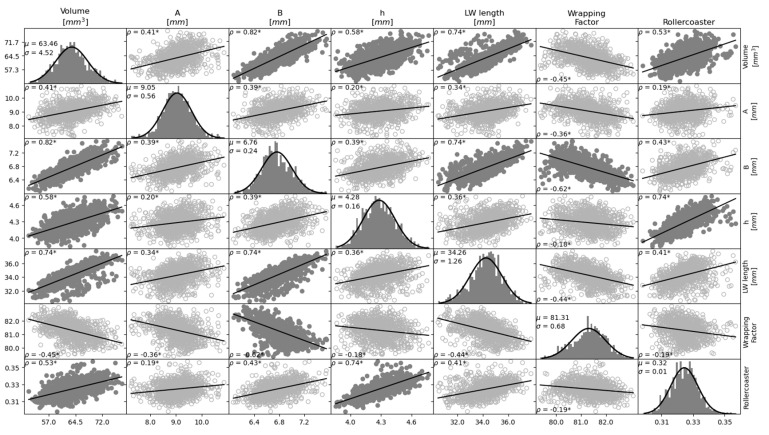
Population statistics and correlation plots between anatomical features of the cochlea. Histograms of the parameters with fitted Gaussian curves are shown in the diagonal of the matrix. Scatter plots show the correlation between the parameter indicated in the column titles and the parameter in the row titles. A strong correlation (ρ > |0.50|) between parameters is represented by scatter plots with filled circles and a weak correlation is shown by empty circles. Solid lines indicate the linear regression curves. Note that the scales of the y-axes do not apply to the histograms. ρ: Pearson correlation coefficient; µ: mean; σ: standard deviation; * depicts a significant correlation (*p*-value < 0.05).

**Figure 3:**
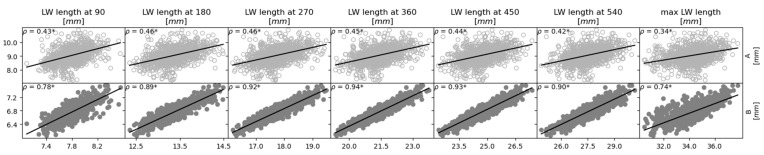
Correlation plots between cochlear duct lengths and cochlear size. A strong correlation (ρ > |0.50|) between parameters is represented by scatter plots with filled circles and a weak correlation is shown by empty circles. Solid lines indicate the linear regression curves. ρ: Pearson correlation coefficient, * depicts significant correlation (*p*-value < 0.05).

Figure 2 shows a matrix of correlations and histograms of the cochlear parameters where the histograms can be seen to follow a normal distribution [30]. Strong correlations were found between the cochlear volume and all other parameters (B (ρ = 0.82, *p* < 0.05), height (ρ = 0.58, *p* < 0.05), cochlear duct length (ρ = 0.74, *p* < 0.05) and roller coaster height (ρ = 0.53, *p* < 0.05)), except for A (ρ = 0.41, *p* < 0.05) and the wrapping factor (ρ = −0.45, *p* < 0.05). Cochlear volume was negatively correlated with the wrapping factor. In addition to the strong correlation with the cochlear volume, cochlear B also showed a strong positive correlation with LW length (ρ = 0.74, *p* < 0.05) and strong negative correlation with the wrapping factor (ρ = −0.62, *p* < 0.05). Unsurprisingly, cochlear B was only weakly correlated to cochlear height (ρ = 0.39, *p* < 0.05) and roller coaster parameter (ρ = 0.43, *p* < 0.05), as these parameters are related to a dimension orthogonal to the plane where cochlear B was measured. In addition to the strong correlation between cochlear height and volume, cochlear height was also strongly correlated with the roller coaster parameter (ρ = 0.74, *p* < 0.05), which was measured in the same dimension. Cochlear A did not show any strong correlation with the other parameters. The correlation between cochlear B and A was also weak (ρ = 0.39, *p* < 0.05). Figure 3 shows the correlation plots between parameters A, B and the LW length at different cochlear angles (90°, 180°, 270°, 360°, 450°, 540°). In general, B shows a stronger correlation to the LW length than A at all angular insertion depths.

**Figure 4:**
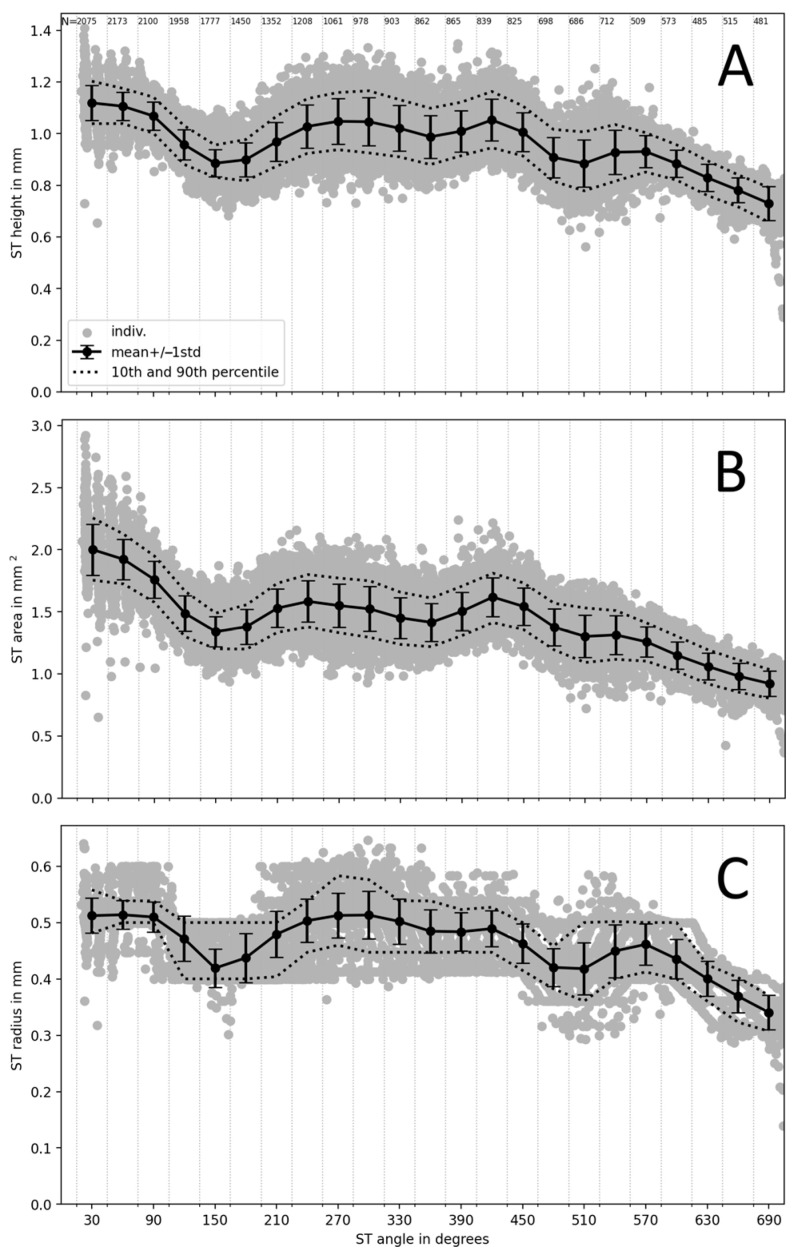
Scala tympani maximum vertical height (**A**), area (**B**) and radius of largest fitted circle (**C**) as a function of the angular distance. Dots represent individual measurement points. Error bars represent the mean and ±1 standard deviation; dotted lines show the 10th and 90th percentiles. Vertical dotted grid lines indicate the angular distance bands that were used to select the N measurement points indicated at the top of panel A to calculate the statistics.

Figure 4 shows the evolution of the ST height, area and radius parameters along the cochlea. All investigated ST parameters show a non-monotonic decrease between 0 and 570° followed by an approximately linear decrease up to 690°. Between 0 and 570°, all ST parameters display notches around 150°, 360° and 510°, and local peaks around 270° and 420°. These could be due to the presence of the porous bone surrounding the common cochlear artery [39].

### 3.2. Inter-Sex Analysis

**Figure 5:**
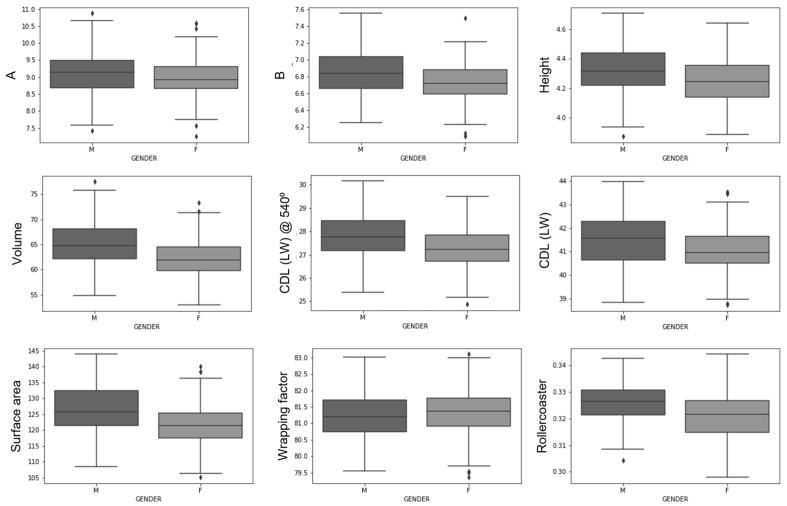
Inter-sex population comparison depicting that female ears are generally smaller and more tightly wound than male ears. CDL (LW): lateral wall cochlear duct length.

Figure 5 shows the inter-sex differences between each cochlear parameter. For the male population, the following dimensional characteristics were observed: A (mean: 9.11 ± 0.58 mm, median: 9.13 mm, inter-quartile range (IQR): 0.81 mm), B (6.85 ± 0.25 mm, median: 6.83 mm, IQR: 0.37 mm), height (4.32 ± 0.15 mm, median: 4.31 mm, IQR: 0.22 mm), volume (64.93 ± 4.40 mm3, median: 64.70 mm3, IQR: 5.99 mm^3^), cochlear duct length (41.48 ± 1.06 mm, median: 41.56 mm, IQR: 1.61 mm) and the wrapping factor (81.20 ± 0.69°, median: 81.19⁰, IQR: 0.97⁰). By comparison, the following dimensions were observed for the female population: A (8.97 ± 0.52 mm, median: 8.92 mm, IQR: 0.63 mm), B (6.73 ± 0.21 mm, median: 6.71 mm, IQR: 0.28 mm), height (4.25 ± 0.15 mm, median: 4.24 mm, IQR: 0.21 mm), volume (62.04 ± 3.91 mm^3^, median: 41.90 mm^3^, IQR: 4.70 mm^3^), cochlear duct length (41.07 ± 0.91 mm, median: 40.96 mm, IQR: 1.13 mm) and the wrapping factor (81.30 ± 0.71°, median: 81.36⁰, IQR: 0.85⁰). An independent *t*-test revealed statistically significant differences for all parameters except the wrapping factor. Generally, female cochleae seem to be smaller and more tightly wound around the modiolus than male cochleae. However, all parameters showed a significant overlap between the two populations.

### 3.3. Intra- and Inter-Patient Analysis

**Figure 6:**
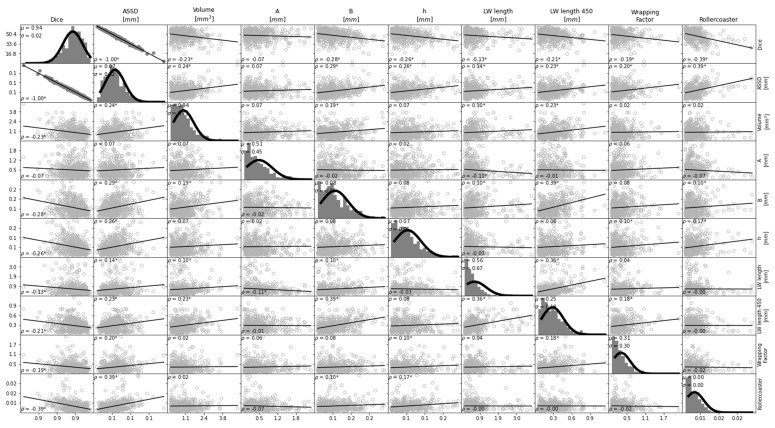
Intra-patient population and comparative correlation plots for imaging and clinical parameters. Strong correlations (ρ > |0.50|) between parameters are represented by scatter plots with filled circles and weak correlations are shown by empty circles. Ρ: Pearson correlation coefficient; µ: mean; σ: standard deviation; * depicts significant relation (*p*-value < 0.05).

The intra-patient analysis yielded mean Dice coefficients of 94.15 ± 0.01% and 91.51 ± 0.02% for cochlea and ST, respectively, indicating high congruency. Similarly, strong correlations were also observed for surface distance metrics (Table 1). The similarity between the cochlea and ST was also high (ρ > 0.97, *p* < 0.05), wherein a strong negative correlation was observed between surface distance errors and Dice coefficients (ρ < −0.99, *p* < 0.05) (Figure 6).

Interestingly, inter-patient analysis also yielded high similarity indexes in terms of imaging analysis, with cochlear and ST Dice coefficients of 93.90 ± 0.05 and 91.04 ± 0.06%, respectively. Statistical analysis revealed no significant difference (*p* > 0.05), even when sub-divided into groups based on cochlear size and shape (A, B, wrapping factor). However, the inter-patient variability was four times the intra-patient variability. Another interesting observation was that there was no correlation between imaging and clinical intra-patient metrics (Figure 6). The only correlations observed were with the roller coaster factor (ρ = 0.45, *p* < 0.05), B (ρ = −0.17, *p* < 0.05) and the wrapping factor (ρ = 0.14, *p* < 0.05).

Concerning the clinical metrics defining the size and shape of the cochlea, intra-patient analysis revealed a mean difference of 0.01 and 0.05 mm for dimensions A and B, respectively, suggesting that neither of the sides is generally larger than the other. By contrast, a mean absolute difference of 0.50 and 0.08 mm was observed for the same parameters. The *t*-test revealed a statistical difference (*p* < 0.05) for most of the clinical metrics, suggesting that size and shape of contralateral ears are not the same. Interestingly, the well-known size metrics A, CDL and volume did not reveal a significant difference (Figure 6).

The difference in B showed medium correlations with differences in cochlear duct lengths (ρ = 0.28–0.54, *p* < 0.05), wrapping factor (ρ = −0.2, *p* < 0.05), volume (ρ = 0.49, *p* < 0.05) and surface area (ρ = 0.56, *p* < 0.05), whereas A only showed weak correlations with cochlear duct length (ρ = 0.12–0.15, *p* < 0.05) and the wrapping factor (ρ = −0.10, *p* < 0.05). The roller coaster factor correlated with the height of the cochlea (ρ = 0.463, *p* < 0.05), whereas the discretized duct lengths showed medium and low correlations with most metrics (ρ < 0.66, *p* < 0.05).

## 4. Discussion

There is a need for large, automated population studies on cochlear anatomy to improve our understanding of the structure and its implications for CI surgery. The goal of this study was to better understand the anatomy of the cochlea and its variability in size and shape, which is important for developing less traumatic electrode arrays and insertion guidance for cochlear implantation surgery. The shape and size of the cochlea can also influence the choice of cochlear implant electrode, with flexible electrode arrays being preferred for more complex cochlear shapes, whilst rigid electrodes are more suitable for cochleae with a more straightforward shape and ossifications. Knowledge of the density and location of spiral ganglion cells can help surgeons choose an electrode array that is most likely to provide good electrical contact with the spiral ganglion cells coupled with minimal frequency mismatch and therefore exhibiting the best hearing outcomes for the patient [41]. In addition, knowledge of cochlear morphology can help surgeons in identifying any abnormalities or variations in the anatomy of the cochlea that may impact on the placement or function of the cochlear implant electrode. By understanding these variations, surgeons can tailor their surgical approach to the specific needs of each patient.

Previous studies have mostly focused on the size, rather than the shape and other parameters, and have only been able to analyze a small number of temporal bones due to the time-consuming nature of manual measurements which limits the scope of the analysis. The use of automated analysis is particularly important in the context of cochlear implant surgery, as manual measurements can be time-consuming and inconsistent. For example, a recent study found that manual measurements of cochlear duct length (CDL) had a maximum absolute intra-rater difference of 3.2 mm and the intra-rater reliability between the two radiological methods used in the study was only 0.65–0.84 [42], indicating that manual measurements may not be reliable. Furthermore, manual measurements were deemed reliable only up to 720 degrees in both CT and MRI scans.

Recent advances in automated analysis tools such as CoreSlicer 2.0 (CoreSlicer, Montreal, QC, Canada), Innersight 3D (Innersight Labs, London, UK), Arterys (Arterys Inc., Redwood Shores, CA, USA), etc., have made it possible to conduct larger studies with more robust and reliable results, as demonstrated in a recent study on cardiac anatomy which showed the feasibility and reliability of using automated analysis tools for population studies [43]; thus, similar approaches can be applied to cochlear anatomy. This study analyzed a large number of clinical temporal bone CT images using Nautilus (v20220801; Oticon Medical [31]) to determine cochlear morphology and characteristics, making it more efficient and robust than manual measurements. Nautilus is a web-based image analysis tool that supports the automatic analysis of pre-operative surgical planning and post-operative assessment for cochlear implant procedures; additionally, ithas the potential to influence the intraoperative workflow in an augmented reality setup and to control insertion forces and trajectories.

The analysis showed that cochlear morphology follows a Gaussian distribution, meaning that most cochleae fall within a typical range of sizes and shapes, with relatively few individuals falling outside of this range. Multiple recent studies have drafted cochlear duct-length prediction models based mainly on dimension A or a combination and dimensions A and B [28,40,44]. Another advantage of using AI-based automatic segmentation tools is that duct lengths can be easily computed in the original image space, decreasing dependence on such mathematical models.

Cochlear dimensions A and B were observed not to be well-correlated with each other. The study also suggests that dimension B is more correlated with cochlear duct lengths, the wrapping factor and volume than dimension A, contrary to popular belief. This suggests that cochlear B may be a more important factor in determining the optimum diameter and length of the electrode array. Moreover, the correlation between cochlear dimensions and discretized duct length increases as the cochlear angle increases, further supporting this observation.

Additionally, the study found that cochleae in female populations tend to be smaller and more tightly wound around the modiolus than male cochleae, but there is a significant overlap between the two populations. There is also a need to study the inter- and intra-individual variability of cochlear anatomy, as this can impact on the reliability of population statistics and the generalizability of findings. Some studies have suggested using contralateral ear CT images when a preoperative CT image for the target ear is not available [45]. However, more research is needed to confirm this and determine the extent of the variability addressed in this study. On average, the dimensions of both ears are similar, but there are statistically significant intra-individual differences in clinically relevant dimensions. This suggests that, while the average size and shape of the cochlea may be similar between the left and right ears, there can be significant differences between the two ears of an individual. However, the results showed that inter-individual variability is four times greater than intra-individual variability, suggesting that contralateral ear CT may be used for analysis only as a last resort if preoperative imaging is not available.

The study also found that the scala tympani size varies considerably among the population, generally decreasing along the insertion depth with dimensional jumps along the trajectory (also observed in a previous study on µCT images [27]). This means that the size of the scala tympani can change significantly as the electrode array is inserted. These findings can help reduce insertion trauma and preserve residual hearing, which, in turn, may impact on the performance of the implant.

In conclusion, the results of the study suggest that certain cochlear parameters are strongly correlated and there are sex-based differences in cochlear dimensions. The results also suggest that it may be necessary to use individualized cochlear models to accurately predict surgical outcomes and optimize implant design. The implications of this research are significant for CI surgery. The size and shape of the cochlea can affect residual hearing, as well as the translocation and tip foldovers/buckling of the electrode array. The mean size and shape of the cochlea, as well as its cross-sectional analysis along the spiral, can provide important information for determining the optimum diameter and length of the electrode array, leading to better hearing outcomes for patients.

## Figures and Tables

**Table 1:** Intra-patient analysis of imaging and clinical parameters. Positive values in the mean column represent a larger right cochlea and vice versa. ASSD: average symmetric surface distance; HD: Hausdorff distance; CDL: cochlear duct length; LW: lateral wall.

Left vs. Right (*n* = 458)	Absolute Mean	Standard Deviation	Mean	Maximum	Minimum
Dice (ST)	91.51	0.02	-	96.34	81.91
ASSD (ST)	0.05	0.01	-	0.11	0.03
HD (ST)	0.34	0.11	-	1.17	0.14
Dice (CO)	94.15	0.01	-	97.17	85.74
ASSD (CO)	0.07	0.01	-	0.15	0.04
HD (CO)	0.39	0.12	-	1.20	0.16
A (ST)	0.50	0.44	−0.03	2.38	−2.32
A (CO)	0.51	0.44	−0.01	2.21	−2.31
B (CO)	0.08	0.05	0.05	0.30	−0.29
Height	0.07	0.05	0.01	0.29	−0.27
Volume	0.95	0.76	−0.06	5.38	−4.85
Surface area	1.66	1.21	0.63	8.39	−5.26
Wrapping factor	0.31	0.29	−0.08	1.58	−2.22
Wrapping ratio	0.44	0.57	−0.01	3.09	−3.31
Roller coaster	0.004	0.004	−0.001	0.01	−0.03
CDL_LW@90°	0.08	0.07	−0.02	0.36	−0.58
CDL_LW@180°	0.10	0.10	0.01	0.63	−0.64
CDL_LW@270°	0.17	0.13	0.06	0.80	−0.73
CDL_LW@360°	0.22	0.17	0.09	0.97	−0.96
CDL_LW@450°	0.25	0.18	0.11	1.14	−1.15
CDL_LW@540°	0.25	0.20	0.08	1.42	−1.19
CDL_LW	0.56	0.66	0.09	3.86	−3.6
CDL@540° approx. [28]	1.61	1.40	−0.05	6.92	−7.23
CDL approx. [40]	0.71	0.61	0.068	3.08	−3.02
Insertion angle@17 mm	3.33	2.70	−1.15	15.32	−14.88
Insertion angle@19 mm	4.57	3.56	−1.81	20.79	−18.81
Insertion angle@21 mm	5.37	4.10	−2.26	24.50	−20.83
Insertion angle@23 mm	6.35	4.75	−2.77	28.17	−26.46
Insertion angle@25 mm	7.53	5.58	−3.11	34.74	−29.05
Insertion angle@27 mm	8.17	6.27	−2.85	35.87	−35.41

## Data Availability

The dataset analyzed within the scope of the current study cannot be made publicly available, as it has been made available to the authors under the specific authorization of CHU Nice. This authorization does not extend to the public publication and distribution of the data. Access to the Nautilus tool is, however, available upon reasonable request at raui@oticonmedical.com.
